# The Role of Midlife Occupational Factors for Trajectories of Cognitive Change

**DOI:** 10.1093/geroni/igaa057.1914

**Published:** 2020-12-16

**Authors:** Gizem Hueluer, Jelena Siebert, Hans-Werner Wahl

**Affiliations:** 1 University of South Florida, Tampa, Florida, United States; 2 Heidelberg University, Heidelberg, Baden-Wurttemberg, Germany; 3 University of Heidelberg, Heidelberg, Baden-Wurttemberg, Germany

## Abstract

Cognitively enriching environments are usually related to higher levels of cognitive performance, while associations with longitudinal change are less clear. In the present study, we used 20-year longitudinal data from the German Interdisciplinary Longitudinal Study of Adult Development and Aging (ILSE) to examine the role of occupational factors for longitudinal trajectories of cognitive function in midlife. To do so, we used data from 374 participants in the ILSE midlife cohort (born in 1950-52; mean age at baseline = 44 years; 44 % women). Our findings showed that cognitively enriching work environments were associated with higher levels of cognitive function at baseline; however, these associations were not independent of control variables including education. There was no evidence that enriching work environments were related to the maintenance of cognitive abilities. In sum, our findings are in line with notions of “preserved differentiation”. We discuss potential mechanisms underlying these findings.

